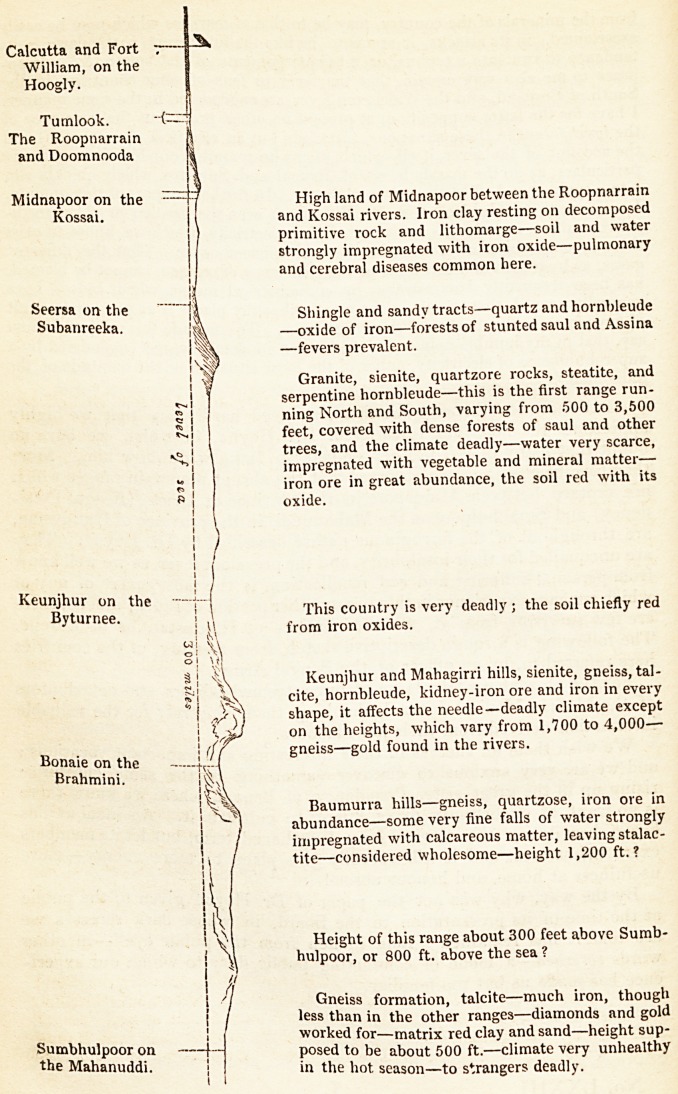# The Madras Quarterly Medical Journal for 1841

**Published:** 1842-07-01

**Authors:** 


### The Madras Quarterly Medical Journal for 1841. Edited by *Samuel Rogers* and *Alexander Lorimer*, M.D. Assistant Surgeons, Madras Establishment.

We are much pleased with Mr. Rogers' and Dr. Lorimer's Madras Medical Journal, and very much pleased with their able and indefatigable contributors—the staff and regimental surgeons of Her Majesty's and of the Indian army—for their statistical reports of corps and stations, and their endeavours to examine and illustrate the influence of tropical climates on European constitutions. In the all-important walks of medical topography and statistics, Madras, we regret to say, stands alone in our Indian empire, in so far as the medical authorities, the Boards, are concerned; and it is but justice to say that to Mr. Annesley is due the introduction of statistical reports in the South of India;—at the same time, we think he would have done better had he at once introduced the system of record so long in use in the Royal Service, as proposed by Mr. Martin in Bengal; for, without uniformity of system in examination, nomenclature, and mode of reporting, comparison—the balance-sheet of the profit account—is wanting to science.

The statistical reports of the troops of the colonies, prepared by Major Tulloch, and printed by order of Parliament, are now standard works of authority in medicine—referred to and quoted by every physician and surgeon, in every quarter of the civilized world, who would analyze and compare facts, bearing on the influence of climate, in its true and extended sense, on military health. The naval reports will command the general attention on like grounds, at the same time that we think their value would have been very greatly enhanced by the adoption of formulæ, similar to those of the Deputy Inspector Marshall and of Major Tulloch, thus facilitating examination and comparison.

We need not say how very much both the Reports last alluded to would have been enhanced also in value, had it been in the power of the authors to exhibit results of equal certainty concerning the native population, civil or military, of the several countries to which their reports refer; and here we would remark with regret on the apathetic neglect of the Indian medical authorities of medical topography and statistics—subjects which we know their leisure, their opportunities for observation, and the institutions of police all over India, afforded them such very ample opportunities of cultivating—yet we have this subject untouched by the authorities, and had it not been for Mr. Martin's plan of 1835, ultimately carried through, he tells us, by the direct Act of the Government, the Empire of the East, in the sense we speak of, would have remained a sealed book to us. It is this we complain of; and, if not for the sake of science, for that of their very seats, we would recommend to certain gentlemen, members of boards, to bestir themselves. Boards are not in high estimation with the European public, whatever they may be in the Eastern hemisphere; and if it be found that they are incapable of following in the march of improvement, even at the respectful distance of 20 years, it may be found out, without much forecast or acuteness of perception, that Boards are in reality screens of the very most useless description.

We know that, shortly after the rejection of Mr. Martin's suggestion for adopting uniformity in reporting with Her Majesty's Service, the medical authorities of Bengal, were, with singular want of candour, patching up the old forms with a view to evade his plan, and deprive him of any credit that might arise from it; but, as might be expected, this patchwork of elementary and ultimate elements, all thrown clumsily together, proved only the incompetence of the framers, and an useless trouble to their subordinates.

At the very time, too, when the Board was thus performing its office of obstruction, its secretary was lecturing the people of India, in the columns of the daily newspapers, on the faults of the judicial system, and dedicating immortal poetry to "*The People of Scotland!!*" This, we say, is too bad.

We do not desire to be severe on persons who have grown old in the practice of doing nothing, and to whom a very feeble exertion, in such a climate, might perhaps be detrimental; but we would ask the opponents of all improvement whether they imagine that the commander-in-chief or the governor-general, who is said to be very partial to the army and its interests, are likely much longer to suffer such a neglect as is here proved. We think not; and we repeat that, for the sake of their seats, if for nothing else, they had better bestir themselves a little; for the public will not now-a-days be satisfied with an enumeration of the numbers of pairs of leather breeches worn out, or of tons of tobacco consumed in smoke by their High Mightinesses of the Board in deliberation, while nothing is done. The cows of New York made the roads while the council was deliberating; and in Bengal, we shall have our moral Sunderbunds cleared by other hands, if our High Medical Mightinesses of the nineteenth century do not order matters to better purpose. We must not allow New York to "progress" so far a-head of Calcutta. But to be serious:—hitherto it has been the boast of the medical profession that, in addition to the just performance of its more especial and honourable functions, it has on all occasions taken the lead in every measure calculated to enlarge the region and the empire of civilized life, reclaiming the savage and the wilderness. We are proud to assert that nowhere has this quality in our profession been more amply displayed than in India, and it grieves us therefore the more to witness its exception there, and in our seniors, whose duties and inclinations ought to prompt them to other and better conduct.

We have now much pleasure in returning to Mr. Rogers and his friends of both services, whom we very sincerely congratulate on the success of their useful proceedings. The general influence of the climate of India and the geography of disease receive much attention in the pages of the Madras Journal; but for the present we will confine ourselves to what is said in the third volume on the Morbid Effects of High Temperature—a most important subject; and if we shall be found to differ somewhat from our authorities, we can assure them we do so in all diffidence and kindness —knowing well the difficulties of the subject. This article is the contribution of Drs. Mortimer, Bisset, M'Grigor, Shanks, and of the Deputy Inspector Murray. It comprises the history of cases, post-mortem examinations, pathological observations, &c., by the several officers named. The subjects of disease were generally troops recently landed ("unacclimated") in India—recruits, volunteers, and sick; and there was in many, if not all the instances, "increased drinking;"—indeed, most of the fatal cases recorded in the 39th Regt. were those of men who had just previously been drinking ardent spirits. "The hot winds," says Dr. M'Grigor, "were nearly of the temperature of 112° Fahr., and caused a constricted feeling in the chest, in those exposed to it, as if of approaching suffocation; and this, together with the impression on the nervous system from the direct heat of the sun, caused a great many men to become ill. It is probable that the blood becomes expanded, and in this way acts like an urgent plethora, while the nervous axes, both ganglionic and cerebro-spinal, are simultaneously impaired."

The regimental reports are closed by "*Clinical Remarks*" from the late talented and experienced Dr. Murray, written just as he had received orders to proceed to Calcutta to succeed Dr. Macleod, the late Inspector General of Her Majesty's Hospitals in India, of whom we had hoped ere now to see some notice commemorative alike of his distinguished military services, and of his most excellent character; but death is so rife with us in India, that men, even the most distinguished, pass away and are soon forgotten.

Dr. Murray begins his remarks by observing on the frequency of death from high temperature amongst British soldiers in India, and states that, in his opinion, the subject requires much more clinical consideration than he gave to it in his former remarks. "The variety of the symptoms arising from this cause, and the confessed imperfect knowledge of their pathology possessed by the profession in general, induce me to direct attention to this point, and to solicit further information respecting such affections from those who may have opportunities of affording it."

After enumerating the semeiology, as collated from the several reports Dr. Murray thus concludes:—"Now there are three principal classes of disease attended with sudden death, viz. syncope, or death beginning at the heart; asphyxia, or death beginning at the lungs; and apoplexy, or death beginning at the brain; between the symptoms of which and the foregoing histories, and the post-mortem appearances in each, it will be most important to draw a comparison, in order to determine the nature of the primary pathological effect of the cause under consideration."

Without stopping to question the justice of the above definitions, we would here call attention to the necessity of separating the effects of ardent spirits, used under a high temperature, from those of direct solar exposure in temperate persons, otherwise we shall be confounding together matters that deserve the most careful and separate consideration. It is quite well understood in the British army in India that, when we speak of a temperate soldier, we merely intend to convey the idea of a man not given to excess; and when we hear another soldier mentioned as "of rather temperate habits," we at once perceive that he may at the same time be in the daily habit of using so much ardent spirits as would very speedily destroy his officer. Now, these two descriptions apply to the very smaller portion of the men who died, as here described, from the effects of high temperature. The rest were men whose habits of life have already been pointed out—in short, men given to excess in drinking.

We do not make these remarks to discourage inquiry—far from it; but we would *direct* inquiry, so as to lead to discriminating and accurate results. We need not remind our zealous and able brethren in the East, of the varied, numerous, and extended examples furnished by the medical history of our army, all over the globe, to shew how great is the exposure, how great and continued the labour under a tropical sun, and how high the temperature that has been sustained with impunity by the British soldier, when under the conservative influence of what Sir John Moore calls the interior economy and discipline in corps; in other words, of well-regulated habits of life. The history of Sir John Moore's hill campaign in St. Lucie—the account given by Dr. Robert Jackson, of the soldiers of the Royal Scots who drained the marsh around Fort King George—the numerous instances given by the same physician of forced labour in the tropical sun by British and French prisoners of war—the marches of Napoleon in Egypt and Italy—the march of Sir David Baird across the desert from Cosseir to Cairo—many of Wellington's forced operations in the Peninsula—the campaigns of Macdonald and Suchet in the Eastern provinces of the same country;—in short, there are examples without number of efforts the most extraordinary, and under the greatest disadvantages, being made by soldiers of various countries without apparent injury to health. The conclusion we would come to then, is this, that we are not warranted in drawing general conclusions from partial instances, and that a more detailed and distinct information is necessary in order to enable us to separate what is due to one cause from what is due to another. Our opinion of the cases related in these reports is, that to heat alone can be referred but a moderate portion of the evil result, and that, without the drink and other stimulation from diet, we should have had but few deaths; in short, that more is ascribed to mere temperature without sufficient reference to other influential causes, than the case warranted. The title of Article V. is therefore, in our opinion, objectionable, because calculated to divert the mind of the inexperienced from the true relation of varied cause to the effect. The power of enduring heat, *under a sudden effort*, or *while the mind is keenly occupied, possessed by British soldiers in*

*India, even beyond the natives,* has often been remarked; and the same quality has been exhibited in other climates, and under opposite extremes of temperature. Our own impression, after a lengthened experience and a careful reference to statistical record, is, that to *long and continued exposure to a high temperature, conjoined to a habit of intemperance,* must be ascribed more than half the deaths that occur amongst British soldiers in India—a strong reason for limiting the period of service in the East, and for reducing it to something like what has been done for the troops in the West.

Observations on the Hill Fevers of the Southern Peninsula of India; with some Remarks on Magnetism and Electricity as a probable Cause of Fever and some other Disorders. By Dr. *Heyne,* Madras. Art. I. No. X.

We have ever considered the geological nature of the soil as one of the most powerful of the causes of physical climate; and in the last edition of the work on Tropical Climates by the senior editor of this Journal, assisted by Mr. Martin, will be found a sketch of our sentiments on that head, referring more especially to the climate of Bengal. In the article mentioned we have referred also to the various supposed sources of the electricity of our atmosphere; but in truth the subject is an obscure one, and in the present state of our knowledge we refer with pleasure to such an ingenious and elaborate article as Dr. Heyne's, were it for no other purpose than to direct inquiry, where facts are so difficult of being obtained.

After enumerating the symptoms of the hill fever, Dr. Heyne observes that the ordinarily received opinions as to the vegetable or marshy origin of fevers will not here hold, for that " the hills are here not more woody than in other healthy places; some indeed, where the epidemic of 1808 and 1810, as well as the endemic, were most destructive, are quite naked of trees, as Diudigal, Madura, and the rocks west of Seringapatam."

"Now, if it should be found, that this fever exists *constantly* and *invariably* among *certain description of hills,* when others of a *different composition* are as *constantly free from the same,* would it not become reasonable to suppose that the *nature* or *composition* of the rock itself must furnish the cause of the calamity?

The hills where it is found to prevail, appear, at first view, to be quite harmless, as they are a *granite,* which is the most common rock-kind on this globe. They contain, however, besides *quartz, felspar,* and *mica,* a great proportion of *ferruginous hornbleude,* which, by its disintegration or separation from the rock, becomes highly magnetic, and *in which,* I suppose, the *cause resides* which produces this fever, besides a great train of other disorders. This iron hornbleude occurs in such quantity, that all rivulets, public roads, indeed, all hollows along these hills are filled with its *sand*; from which, also, all the iron in this part of the country is manufactured. This granite is remarkable for its disintegration, as it not only separates during the hot season in large masses of many tons, but crumbles as easily into its composing particles, and is found as sand in great abundance, not only near every rock, but near every stone, from whence it is carried by the torrents during the rains to the lower parts of the country, and thus forms the particular mark by which these hills may be distinguished from all others. It is generally not attracted by the magnet *when united* to the mass, even when it occurs as in hornbleude state, or greenstone, in the greatest abundance, but after *it has been separated* it is attracted as much as any iron filings. This may be owing to the incipient state of oxydation, or more likely, to the development of magnetism by the high temperature to which it has been exposed in the hot season, which also may have weakened the cohesion of the rock, and caused its disintegration in the mass.

Hills of this description form the principal ranges of the Ghauts, as far at least as the Godavery; they predominate also among the smaller, and in single hills and rocks in the low country, so that they might be taken for the exclusive rock formation of this country. However, fortunately, this is not quite the case. They are easily recognised at a distance by their very rugged and abruptly pointed appearance, and the great steepness at their tops. The ranges of this formation are also very interrupted, and generally consist of rows of single hills, although to the Southward, I have found them also connected at bases, and in triple and quadruple ranges."

Dr. Heyne then gives an excellent topographic description of the hills "which have rendered themselves known to Europeans for the malignity of the fever," and after that of such as are "*as constantly free of the hill fever.*" This is the right kind of topography, but for obvious reasons we cannot here enter into it. The hills where the fever is "totally unknown," Dr. Heyne describes as "*primitive* trap, which consists of *quartz, felspar,* and REAL hornbleude." He then adds that the epidemic fever of 1808 stopped short at a range of hills of this latter composition, in the Coimbatore district—a remarkable fact.

"These two ranges of trap proceed with very little or no admixture of iron stone through the whole Baramahal, from Namcul to Darampoory and Vellore; the rocks are sometimes compact hornbleude and greenstone, or basalt, all belonging to the same formation; but here and there hills appear among them of iron granite, which stand in connexion with other ranges of that description in that province, both East and West of that valley, which have the hill fever as virulent as in other parts of the country, where whole ranges of these hills occur.

A most remarkable instance illustrative of the above facts, and of my deductions from them, I found at Tripatoor, which lies in the above valley, close to a large table-land, the rock of which is sand-stone. I asked there a respectable native whether any such disorder as fevers, were frequent in the country, but received in answer, 'No, thank God, not within ten miles of this place; at Javadymalle, a hill fort where no man can live two days without getting it.' To this place a peon was despatched with the simple order of bringing two or three stones from the rock of the hill, and some sand as might be found on the road. The man returned, and brought pieces of a rock composed of red felspar, quartz, and plenty of ferruginous hornbleude; and the sand of the road consisted entirely of magnetic sand and particles of felspar.

I must name now the Pulicat hills, among which, as far as they extend to the Southward (Chittoor) the hill fever is totally unknown. I was particular in my inquiries on this subject, in the beginning of this year, when among them. They consist entirely of flinty slate, and are bare in some places as they are woody in others, and as lofty and as low as the granite hills.

I come now to a country and hills where I have lived myself for some years, the Cuddapah District. It is divided from Gurrumcondah on the South, and from iron granite and the hill fever, by a range of flinty slate. The same bends there to the northward, where the ranges thicken as they advance, and leave narrow valleys as far as Cummuur, and further up the river Kishna. The whole or most of these hills belong to the clay-slate formation, some are calcareous, all however are free of the hill fever. Other fevers may occasionally be seen, such as simple intermittents and bilious remittents, but they do not, like the hill fever, run into a typhus, and the cautious may easily guard against and get rid of them.

This is the largest extent of inland country which I know to be free of the hill fever, viz. from Cuddapah to Kishna near Chintapilly, a place that has been at all times dreaded for its fevers. There, the iron granite hills prevail again. To the westward of Cuddapah, the healthiness of the country extends to the Ganjecottah hills, which belong to the flætz trap formation, consisting of sandstone, limestone, jasper and hornstone pebbles cemented together, and which are perfectly free of magnetic ironstone.

Bababudden is another range of hills which is remarkably free of hill fevers, although it lies between places of notoriety for such, as Seringapatam to the S.W. and Chittledroog to the N.W. and Naggury to the W. an unwholesome country amongst the Ghauts. It belongs to the *clay-slate* formation, and *active magnets* are found in large depositions on them. It rains on them for six months in the year continually, when plants keep fresh and alive in the open air for many days after they have been taken out of the ground, or broken off the stem. In fact, my observation, viz. that the hill fever on this coast exists *exclusively* among the hills of the granite formation, or where iron-stone is found in large quantities, will be confirmed, the more it is brought to the test.

A principal question arises now, but which, and the answer to it, I presume will be anticipated by every medical man, viz. what can be the particular principle in that rock which should have so powerful an effect on the human frame? I readily ascribed it to the *magnetic* or *electric fluid*, which seems to exist in the greatest abundance in the iron hornbleude, and is disengaged in great quantity, in the hot season.

The electric and magnetic fluids are modifications of each other—a principle now pretty generally admitted. It exists in the air, and that it does in the earth and in the minerals, need scarcely be mentioned, nor are the animal and vegetable kingdoms less indebted for its influence, indeed it is the *anima mundi*. It can be accumulated under certain circumstances in the air, and there is no doubt, that as in magnetism, so it is in iron, and in some other minerals; and as it is elastic, it can be also dissipated from the place in which it is confined. Of course where magnetic iron abounds the electric fluid, whether in its positive or negative quality, will make, under favourable circumstances, its escape.

This must be on common physical principles the case, *when the temperature is more than usually increased;* the hottest season therefore when the rocks exposed to the meridian rays of the sun are raised to the accumulated heat of 220°, is the epoch when the fever rages most, (which we suppose to originate from the greatest development of magnetism). It is known that a high degree of electricity can be raised, in certain minerals by heating them merely, and according to my experiment, the hornbleude which is found in this granite becomes magnetic on being heated, which before shewed no magnetism whatever. It stands also to reason, that the first rain which cools the atmosphere down to 74° must put a stop to the discharge of that principle, and to the farther cause of the fever, for 'cessante causa tollitur effectus.'

It is generally believed, that so powerful a principle has, or must have a great influence, on the animal constitution, although electricity has hitherto been tried, but with very partial success, as a remedy against some disorders; and if I am not mistaken, with more where it has been *abstracted,* where sparks have been elicited, than where they have been imparted. Magnetism has also been tried, but oftener ridiculed by the medical world in England, particularly that which is called animal magnetism.

In my humble opinion it is here the particular magnetism or electricity of the iron granite, without however attempting to determine whether it is the vitreous or resinous; for hornbleude in primitive trap contains nearly as much iron as that of the granite; the iron also in other minerals, as in the magnetic iron slate of Bababudden, and the carbonated iron ores of that country, possesses as much magnetism, even in its active state, yet do they not prove themselves in the least hurtful to our constitution, as that of the iron granite hills; of course if it be electricity at all (as it should appear) it must be that particular modification of it, which is inherent to the iron sand of the granite of this country.

It has been observed by some practitioners, (Mr. Scarman) that the night air in those places, where such fevers occur, is particularly to be dreaded. This seems to militate against the new doctrine, but is actually in support of it; for electricity, as is well known, can be confined to clouds for a considerable time, or can be kept at a certain spot by attraction (as in the ignis fatuus), and of course the same principle, under a different form, but from similar causes, may be kept floating in the air for some time at the particular spot where it has been discharged, and, if it should remain till night, it must be condensed by the coolness of it, and hence will be imparted, or come concentrated to those who expose themselves to it at the time.

The natives are particularly fond of sleeping in the open air with a very slight or no covering, hence one cause of their being oftener subject to those fevers than Europeans.

A moist atmosphere destroys electricity (to use the common phrase) or abduces it; it is therefore but natural, that the first strong rain in the season, besides the cooling of the rocks, should remove the sickness which is the consequence of it; on that account also, in a season prior to the hot, (in January and February) the fever has been restrained by the same circumstance. The heavy dews, among our Ghauts, which some have even considered as the forerunner, or as a powerful cause of these fevers, have absolutely retarded or prevented them. For it should be known and remarked, that these months are reckoned the safest to venture among the Ghauts and to remain there."

"It may be observed further, that all epidemics in this country are preceded by uncommonly heavy rains and some lightning: such was not only the case in the fever epidemy of 1808 to 10, as already said; but such existed before the appearance of the present cholera morbus in Bengal, and now at Madras. I do not suppose, however, that they are in consequence of the rain after it has fallen, and the inundations which have followed it, but from the superabundance of *electric* matter which caused the rain, or in fact from the *same cause* (electricity) derived from a different source.

I would advise, as a precaution, to avoid if possible the living near a hill or rock about which a quantity of magnetic iron sand is found. The distance of two miles would be quite sufficient in common cases, as it has been observed even at Courtallum, where the village, that had suffered much from the fever, has been removed with the best effect to that very distance.

I could now close my writing as I have said nearly every thing which I know at present on the subject, but I will suggest a few hints, which strike me, will not inaptly come from me, although I am convinced they would soon occur to others, and would be most likely better expressed.

It appears in the first instance to me probable, that electricity in general, is the principle which has most influence on our health, and on our life. We live in it constantly, it penetrates every thing, it is as a constituent of every thing, &c. &c. It may abound in some situations,—it may be deficient in others, each of which, must have peculiar effects; the positive or vitreous, the negative or resinous may predominate; either must have its *peculiar* influence. In the preceding pages we have seen what effect it has when it occurs in great quantity from magnetical iron stone (I believe the resinous), it is probable that it may have similar consequences from whatever other sources it may be derived. The fever in the Northern Circars, although it might not be owing to the electricity from the minerals of the country, may be to that of marshes which may be easily ascertained; in its attacks, it seems to be like the hill fevers, particularly in its tendency to run into a typhus, or into enlargements of the spleen, &c. It appears to me also very certain, that the fever in fens of some countries, in the South of England, and the Walcheren fever, are engendered in the same manner. I have for the latter supposition, at present no other proof but the frequency of the *ignis fatuus* in these situations, (certainly but an electrical phenomenon) and the account of the fever itself, which seems to resemble our hill fever in many particulars, as do the marsh fevers of Bengal and Sumatra, which quickly run into a typhus, and affect the spleen violently. In further support of this opinion, I will say, that Abbé Nollet, or Dr. Wilson, or even the gentlemen of the medical committee, have long ago suspected that electricity, which we know to exist there in some abundance, must be the real efficient cause. That the different gases, as hydrogen and carbonic, or the deficiency of oxygen, cannot be blamed, has been frequently demonstrated by eudiometrical means, which indeed have generally proved, that the air in the most unhealthy places is as pure and as full of oxygen as in the most salubrious situations. To conclude this subject, I must say, that in my humble opinion *all fevers* are in some degree engendered by a superabundance of electricity, either of the local situation or the habitude of the individual."

After these lengthened quotations, we need hardly say that we highly appreciate the laborious research of Dr. Heyne, in which we have no doubt that many of our brother officers in India will follow him. More than twenty years ago, we observed that the abrupt mountain ranges which are crossed between Midnapore in the province of Orissa (Bengal Presidency) and Sumbhulpore on the Mahanuddi, in the province of Gundwana, are throughout of the ferruginous nature described by Dr. Heyne. They are unequalled for their insalubrity, and the prevalent fever, as we well know from personal suffering and sad recollection, is the most severe of any of which we have experience in that or any other portion of India; indeed, there are few survivors from it, whether natives of Hindustan or of Europe. The following is a rough descriptive sketch, from memory, of the countries here referred to, by an officer of the Bengal Army.

For the present we take leave of our esteemed editors and contributors of the Madras Medical Journal, thanking them sincerely for the valuable information derived from their joint labours.

We wish them all success in their interesting and important vocations; and we are very anxious to discover something of the same enterprise rising up in the great sister Presidency of Bengal, where we know there is no want of opportunity or of talent to cultivate it. An incubus has borne heavily on the service for a lengthened term, but let its members exert themselves, and it will speedily give place to more energy—more usefulness at home, and honour abroad.

By the way, why was not the paper of Dr. Heyne given to the public at the time of its presentation to the Board, in whose dark recesses we are told it has remained "long hidden from the public eye"—in other words *screened*—a mode of conducting public duty to which our experience has made us but too familiar.

Calcutta and Fort William, on the Hoogly.

Tumlook. The Roopnarrain and Doomnooda

Midnapoor on the Kossai.

High land of Midnapoor between the Roopnarrain and Kossai rivers. Iron clay resting on decomposed primitive rock and lithomarge—soil and water strongly impregnated with iron oxide—pulmonary and cerebral diseases common here.

Seersa on the Subanreeka.

Shingle and sandy tracts—quartz and hornbleude —oxide of iron—forests of stunted saul and Assina —fevers prevalent.

Granite, sienite, quartzore rocks, steatite, and serpentine hornbleude—this is the first range running North and South, varying from 500 to 3,500 feet, covered with dense forests of saul and other trees, and the climate deadly—water very scarce, impregnated with vegetable and mineral matter—iron ore in great abundance, the soil red with its oxide.

Keunjhur on the Byturnee.

This country is very deadly; the soil chiefly red from iron oxides.

Keunjhur and Mahagirri hills, sienite, gneiss, talcite, hornbleude, kidney-iron ore and iron in every shape, it affects the needle—deadly climate except on the heights, which vary from 1,700 to 4,000—gneiss—gold found in the rivers.

Bonaie on the Brahmini.

Baumurra hills—gneiss, quartzose, iron ore in abundance—some very fine falls of water strongly impregnated with calcareous matter, leaving stalactite—considered wholesome—height 1,200 ft.?

Height of this range about 300 feet above Sumbhulpoor, or 800 ft. above the sea?

Gneiss formation, talcite—much iron, though less than in the other ranges—diamonds and gold worked for—matrix red clay and sand—height supposed to be about 500 ft.—climate very unhealthy in the hot season—to strangers deadly.

Sumbhulpoor on the Mahanuddi.

## Figures and Tables

**Figure f1:**